# Melatonin improves spermatogonial stem cells transplantation efficiency in azoospermic mice

**Published:** 2014-02

**Authors:** Mohammadreza Gholami, Ghasem Saki, Masoud Hemadi, Ali Khodadadi, Javad Mohammadi-asl

**Affiliations:** 1 Department of Anatomy, Lorestan University of Medical Sciences, Khorramabad, Iran; 2 Physiology Research Center, Ahvaz Jundishapur University of Medical Sciences, Ahvaz, Iran; 3 Fertility and Infertility Research Center, Ahvaz Jundishapur University of Medical Sciences, Ahvaz, Iran; 4 Department of Immunology, Ahvaz Jundishapur University of Medical Sciences, Ahvaz, Iran; 5 Department of Medical Genetics, Ahvaz Jundishapur University of Medical Sciences, Ahvaz, Iran

**Keywords:** Cryopreservation, Melatonin, Spermatogonial stem cells, Transplantation

## Abstract

***Objective(s):*** Transplantation quality improvement and reduction of cellular damage are important goals that are now considered by researchers. Melatonin is secreted from the pineal gland and some organs such as testes. According to beneficial effects of melatonin (such as its antioxidant and antiapoptotic properties), researchers have proposed that the use of melatonin may improve transplantation quality. The aim of this study was to investigate the effects of melatonin on the spermatogonial stem cells transplantation in the azoospermic mice.

***Materials and Methods:*** The testes of the BALB/c mice pups (6-day-old) after vitrified-thawed, were digested with enzymes (collagenase, DNaseΙ, trypsin-EDTA) to disperse the cells. The SSCs, type A, were isolated from the rest of testicular cells by MACS. Spermatogonial stem cells were labeled with PKH26 fluorescent kit. Labeled spermatogonial stem cells were transplanted into the testes of infertile mice (busulfan 40 mg/kg). The mice died two months after transplantation and the efficiency of spermatogenesis was investigated. TNP2 and hematoxyline-eosin staining were used to detect the efficiency of cell transplantation.

***Results:*** TNP2 were detected in the samples that received melatonin and spermatogonial stem cells transplantation, simultaneously. TNP2 was not detectable in the transplant recipient mice that received placebo for 10 weeks (control group). According to hematoxyline-eosin staining, melatonin improved structure of testes.

***Conclusion:*** Administration of melatonin (20 mg/kg) simultaneously with transplantation of spermatogonial stem cells in azoospermia mouse testis increases the efficiency of transplantation and improves structural properties of the testes tissue.

## Introduction

Spermatogenesis is a complex phenomenon in which proliferation and differentiation of spermatog-onial stem cells continuously seek to become mature sperm (1, 2). Spermatogonial stem cell transplant-ation is a technique in which the spermatogonial stem cells, type A, from a donor animal are injected into seminiferous tubules or rete testes of the recipient testis (azoospermic mice) (3, 4). Following the transplantation, spermatogonial stem cells migr-ated to basal compartment of seminiferous tubules via unknown mechanisms and resume spermato-genesis (4, 5). In animal models (rodents, pigs, goats and dogs), spermatogonial stem cell transplantation into the testes of infertile males can lead to the reoccurrence of spermatogenesis (6-14). The current challenge concerning spermatogonial stem cells tran-splantation is increasing the quality and safety of cellular transplantation (1). The application of this technique to children rescued from chemotherapy because of lower success in animal models (e.g. mice, rats, goats, pigs and dogs) don’t extend (1). However, the recipient mice were able to reproduce live offspring but litter sizes were reduced in comparison with control group and the concentration and moti-lity of sperms were decreased (15-17). Improving the quality and reducing the harm to transplanted cells during freezing-thawing and recovery of sperm-atogenesis after transplantation of cells seem to be essential (1). Researchers have suggested that melatonin could be used to improve the quality and performance of organ transplantation (18). Use of melatonin in organ transplantation due to its pro-perties such as antioxidant, antiapoptotic, antibiotic, antiviral and immunosuppressive effects appears to be effective. (19-21). Melatonin is small biological molecule that is secreted from pineal gland and other organs e.g. retina, testis (19, 22, 23). Effects of melat-onin are studied in many regulatory functions of the cells such as immune response, cell signaling, protec-ting fatty acids from oxidation and nuclear DNA from damage, controlling the tumor growth and inhibiting cell proliferation, oncostatic action, antiapoptotic effect on many normal cells, enhancing apoptosis in the tumor cells and significant anti-aging properties (19, 20, 22-32) . Effects of melatonin on cells transp-lantation are not clear. It is not clear whether melato-nin can improve spermatognial stem cell transplant-ation efficiency or not. The aim of this study is assessment of concurrent administration of melato-nin with spermatogonial stem cells transplantation in azoospermic mice.

## Materials and Methods

T All experiments were performed in accordance with principles of laboratory animal care. Male 6- day-old- BALB/c mouse pups (N=80) were obtained from physiology research center. Mice were eutha-nized by excessive doses of ketamine HCl (80 mg/kg) and xylazine (10 mg/kg) (Pharmacia and Upiohn, Erlangen, Germany) (21) in accordance with the protocols approved by Ahvaz Jundishapur University Medical Science Animal Care and Use Committee. Testes were vitrified and thawed according to Gholami *et al* methods (33). Testes were transferred to vitrification solution 1 (V.S 1), 2 (V.S 2) and 3 (V.S 3), respectively (Table 1). Finally, samples were transferred to liquid nitrogen tank.

***Thawing procedure***

Samples were maintained for 30 seconds at room temperature and were hold in water bath 37°C until defreeze then, samples were transferred to thawing solution1 (T.S.1) containing 0.5 molar sucrose at 4°C. After 5 min, samples were trans-ferred to thawing solution 2 (T.S.2) containing 0.25 molar sucrose at 4°C. After 5 min, samples were transferred to thawing solution 3 (T.S.3) containing 0.125 molar sucrose at 4°C (33).

***Digestion of 6-day-old mouse testes***

The cells digestion was done according to Milazzo *et al*, with little modification (34). Briefly, after removal of tunica albogina, 6-day-old mice testes were digested in the two steps. In the first step, 10 testes were incubated in 1 mg/ml collagen-ase type ΙV and 200-700 µg/ml DNaseΙ for 15 min at 37°C with slow pipetting. After centrifuging at 100 g for 5 min, in the second step supernatant was discarded and cells were resusp-ended in 1 ml trypsin-EDTA (sigma) and 200 µg/ml DNaseΙ for 5 min at 37°C. Trypsin was inactivated with adding 10% FBS to cell suspension.

**Table 1:** Details of the solution used for vitrification

	0.5 molar sucrose	Ethylene glycol	DMSO	20% FBS	Time
V.S. 1	+	+ (7.5%)	+ (7.5%)	_	10 min
V.S. 2	+	+ (15%)	+ (15%)	_	10 min
V.S 3	+	+ (15%)	+ (15%)	+	10 min

***Separation and purification with Laminin and MACS***

Petri dishes (60 mm) were incubated with 20 µg/ml laminin, overnight. Supernatants were removed and Petri dishes containing laminin were washed with PBS buffer. Petri dishes were incubated with %0.5 mg/ml BSA, for one hour at 37°C to prevent nonspecific bindings and then they were washed with PBS buffer (35). In next steps, spermatogonial stem cells were purified with CD90.1 antibody. The cells, digested in the previous step, were incubated for 1 hr in the Petri dishes containing laminin at 32°C. Next, Petri dishes were washed with PBS. Cells that were attached to laminin were isolated by using trypsin - EDTA. CD90.1 (Thy1.1^+^) was used to detect spermatogonial stem cells type A. The procedure was performed according to manufacturer manual (Miltenyi Biotec, order no. 130-094-523). In brief, 10^7^ of total cells were centrifuged at 300 g for 10 min. Cell pellet was resuspended in 90 µl of buffer .Buffer solution contained phosphate-buffered saline (PBS), PH 7.2, 0.5% bovine serum albumin (BSA), and 2 mM EDTA by diluting MACS BSA stock solution (# 130-91-376) 1:20 with autoMACS rinsing solution (# 130-091-222). Also, 10 µl CD90.1 microbeads was added. Then, it was well mixed and incubated for 15 min in the refrigerator (2-8). Cells were washed by adding 1-2 ml of buffer and centrifuged at 300 g for 10 min. Next, up to 10^8^ cells were resuspended in 500 µl of buffer. Then, the cell suspension was loaded onto a MACS column, which is placed in the magnetic field of a MACS separator.

***Labeling cells for transplantation***

 Cells were labeled with real fluorescent cell linker kit according to manufacturer catalogs. In brief, cells were centrifuged at 400 g for 5 min. Dye was prepared at the concentrations of 16 ×10 ^-6 ^M and cells were incubated with this dye for 2-7 min at 25C˚. Labeling procedure stops by addition of FBS. Subsequently, cells were washed 3 times in DMEM and kept on ice until transplantation.

***Azoospermic mice for cells transplantation***

Busulfan, 40 mg/kg, was injected intraper-itonealy to mice for 4-6 weeks. The mice will be ready for transplantation, 2.5 months after injection. The male BALB/c mice in our study, were divided into three groups. The first group (n=5) received transplanted cells and 10 weeks melatonin (20 mg/kg). The second group (n=5) received transplanted cells and 10 weeks placebo. The third group (n=5) of mice that did not receive transplanted cells and were injected with melatonin or placebo.

**Figure 1:**
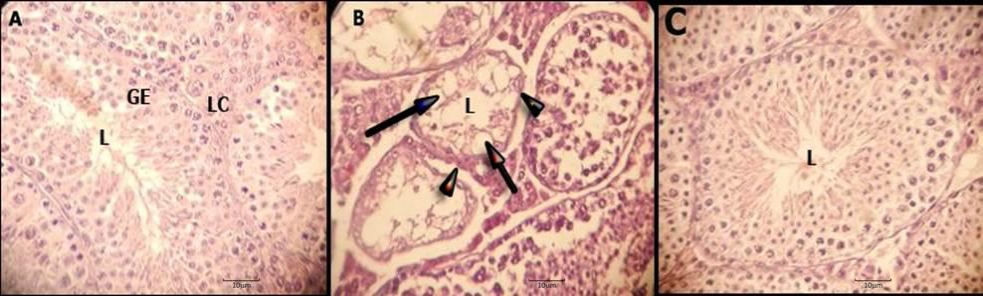
Testis sections stained with hematoxylin - eosin. Section A, samples that received melatonin and spermatogonial stem cells transplantation. Section B, samples that did not t receive melatonin and spermatogonial stem cells transplantation. Section C, samples that received placebo and spermatogonial stem cells transplantation. In the section B, vacuole is shown with arrows and thickening of the basement membrane is shown with arrow heads. Lumen (L), germinal epithelium (GE), Leydig cells (LC). Hematoxylin -eosin. Magnification X 400

***Spermatogonial stem cell transplantation***

 Spermatogonial stem cells transplanted to left rete testes by microinjection, using a needle under a stereomicroscope with the appropriate diameter, after anesthetizing the mice (36). Melatonin, 20 mg/kg daily, was injected intraperitoneally to mice for 10 weeks after transplantation.

***Hematoxyline and eosin staining***

After 10 weeks of spermatogonial stem cells transplantation, the mice were killed with a high dose of anesthetic and testicular tissue was extracted for analysis. Left testes after fixation, embedded in paraffin, cutting and dehydration stained with hema-toxylin-eosin dye for evaluation of histological changes. The results were analyzed based on a descriptive method and the blind evaluation of tissue sections within similarly staged seminiferous tubules was done by specialists.

***Immunohistochemistry***

The replacement of histones with protamine is done via transition nuclear protein 2 (TNP2) in the late stages of spermiogenesis. TNP2 appears in the nuclei at step 10 spermatid. Tracing this protein could be a good indicator to show spermatid. Immu-nohistochemical staining was performed according to the method of Zheng *et al*, with slight modific-ations (37). After fixation, embedded in paraffin and cutting, paraffin was removed from the the specim-ens by Xylene and specimens were dehydrated in ethanol. Antigens retrieval was done in citrate buffer (pH = 6) for 15 min at 98°C. Samples were incubated with PBS-0.3% Triton X-100 with 10% Normal donkey serum for 3 hr. The samples were incubated overnight with primary antibody (Polyclonal IgG Goat). Primary antibodies were diluted in PBS-TS at the ratio of 1:100. At this stage, PBS-TS only was added to the negative control samples. Secondary antibody was diluted at the ratio of 1:50 in PBS-TS. After washing, samples were incubated with DAPI for 12 min. The results were analyzed based on a descriptive method and the blind evaluation of tissue sections within similarly staged seminiferous tubules done by specialists.

***Statistical analysis***

Results (seminiferous epithelium) of treated group with melatonin and control group (untreated group) were compared with Mean-Whitney U-test and SPSS.16 software. Results are presented as MeanSD and statistical analysis were considered significant at *P*_v _=0.001.

## Results

***Study of tissue sections after transplantation using hematoxylin – eosin staining***

 The results were analyzed based on Johnson’s method (38) and the blind evaluation of tissue sections was done by specialists. Statistical semin-iferous epithelium analysis between treated group with melatonin (7.951.85) and control group (1.500.513) according to Johnson's method is significant (*P*_v_=0.001). Tissue sections of the first group that received transplanted cells and melat-onin, showed that large number of sperm was found in the lumen of seminiferous tubes and completes spermatogenesis. Germinal epithelium seminiferous tubes are stratified (Figure 1, section A). Basement membrane is organized and integrated (Figure 1, section A). Leydig cells in the interstitial space are evident (Figure 1, section A). The tubes have a significant thickness of germinal cells and various germ cells were observed including spermatogonia, primary spermatocytes, secondary spermatocytes, round spermatid, spermatozoa and sertoli cells (Figure 1, section A). In the interstitial tissue, visible loose connective tissue with blood vessels, nerves and leydig cells with round nuclei were observed (Figure 1, section A). Tissue sections of the second group that received transplanted cells and placebo, showed that lumen of seminiferous tubes represented a few elongated spermatids (Figure 1, section C). Epithelium of seminiferous is stratified and normal (Figure 1, section C). Basement mem-brane is normal (Figure 1, section C). Leydig cells are showed in the interstitial space (Figure 1, section C). Tissue sections of the third group that did not receive transplanted cells and placebo, showed that lumen of seminiferous tubes are devoid of spermatid and epithelium seminiferous tubes are irregular and destructed (Figure 1, section B). Vacuoles are seen in the epithelium seminiferous tubes and the basement membrane thickening is irregular (Figure 1, section B). Interstitial space is irregular (Figure 1, section B). The destruction of spermatogenesis can be seen clearly (Figure 1, section B). 

Immunohistochemical results for review TNP2

**Figure 2:**
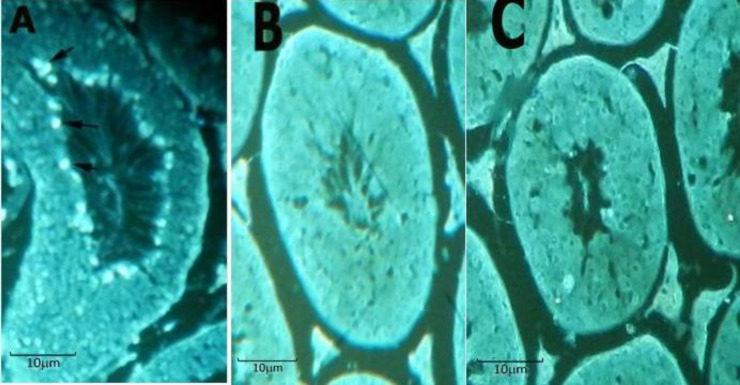
Immunohistological staining for detected TNP2. TNP2, Bright spots in periphery of the central lumen (arrows), detected in section A. Section A, samples that received melatonin and spermatogonial stem cells transplantation, simultaneously. Section B shows the negative control group. Section C, samples that received placebo and spermatogonial stem cells transplantation. TNP2 was not detected in the section C. Magnification X 400

TNP2 is shownin the central part of seminiferous tubules (Figure 2, section A) in the mice that received melatonin and spermatogonial stem cells transplantation (group 1). TNP2 was not detectable in the transplant recipient mice that received a placebo (group 2) for 10 weeks (Figure 2, section C).

## Discussion

Researchers showed that administration busulfan 40 mg/kg to mice lead to complete loss of spermatogenesis (39). Cryopreservation of sperma-togonial stem cells can preserve fertility in children with cancer. Melatonin has antioxidant and anti-apoptotic properties (22). Melatonin seems to be useful in preventing transplant rejection (19). Melatonin can easily cross the cell membrane because of its small size and high lipophilic properties. Very high concentrations of melatonin in the cell nucleus protects DNA against damaging agents (22, 40). Therefore, in this study the effect of melatonin on transplanted spermatogonial stem cells in mice were studied and the results showed that melatonin improves the maturation and survival of spermatogonial stem cells in the recipient testes. Aziz *et al*, showed that administration of melatonin was more effective than stem cell therapy in busulfan-treated mice (41). Aziz *et al*, showed that administration of melatonin to azoospermic mice leads to complete regeneration of germ cells with appearance of elongated and round spermatids. In the stem cell-treated mice, germ cells showed partial or incomplete regeneration with invisible round and elongated spermatids (42). Results obtained by Aziz *et al*, are consistent with the results obtained in this study. Simultaneous injection of melatonin and busulfan reduces the effects of busulfan on spermatogenesis (42). Melatonin administration red-uced testicular damage in the rats that were treated with cisplatin (43) and streptozotocin (44). Researchers showed that melatonin decrease germ cells apoptosis and cadmium-induced testicular stress in testes (45). Administration of melatonin to older men and women may help repairing oxidative damage of gunine DNA (46). Administration of high doses of melatonin (100 mg/kg) protects the testicular against the harmful effects X-rays (47). Melatonin successfully increases the quality of life in patients with tumor (48, 49). This evidence indicates protective effects of melatonin which is consistent with the results of this study. The amount of apoptotic cells in the group receiving melatonin was reduced as compared with the control group. These results confirm finding of other researchers about the anti-apoptotic effects of melatonin. Hemadi *et al*, showed that daily administration of melatonin to mice that received ectopic testicular transplantation for 2 months at the dose of 20 mg/kg led to the resumption of spermatogenesis, decreased apoptosis and improved transplantation (21). Researchers suggested that antioxidant and anti-apoptotic prope-rties of melatonin can protect sperm, epididymis and seminal vesicle (50). D'Istria *et al* showed that melatonin has anti-proliferative effect on germ cells (51). Researchers have shown that melatonin has antiproliferative effect on human prostate epithelial cells and neuroblastoma cells (52, 53). In our previous studies, we showed that supplementation of vitrification-thawing media with melatonin does not protect testicular tissue from injury (33) and melatonin may cause apoptosis in cells that were damaged in the process of freezing – thawing (54). Researchers showed that short-term administration of melatonin was beneficial and side effects have not been reported (19). Researchers suggested that melatonin strengthen the immune system (19). Melatonin role in organ transplantation is still not well understood. Jung *et al*, showed that the administration of melatonin can prevent rejection of transplanted hearts (18). Taken together, it can be said that melatonin may improve the quality of transplantation. In order to examine the effect of melatonin on the process of cells transplantation and to determine the exact mechanism on sperma-togonial stem cells transplantation more closely, we suggest that more studies should be done.

## Conclusion

Simultaneous administration of melatonin and spermatogonial stem cells transplantation can improve spermatogenesis quality. 
